# Disorders of organic acid metabolism and epilepsy

**DOI:** 10.1186/s42494-024-00167-2

**Published:** 2024-08-20

**Authors:** Yuqing Shi, Zihan Wei, Yan Feng, Yajing Gan, Guoyan Li, Yanchun Deng

**Affiliations:** 1 Xi’an Medical University, Xi’an, 710021 China; 2 Department of Neurology, Xijing Hospital, Fourth Military Medical University, 127 West Changle Road, Xi’an, 710032 China; 3 Xijing Institute of Epileptic Encephalopathy, Xi’an, Shaanxi 710065 China

**Keywords:** Organic acids, Epilepsy, Gene genetic variants, Diagnosis, Treatment

## Abstract

Epilepsy can be caused by a variety of causes, such as inborn errors of metabolism, organic acid disorders are the most significant type of metabolic disorders that cause seizures. The clinical manifestations of these diseases are generally nonspecific, and the types of seizures are different. Screening for multisystem clinical symptoms and identifying the underlying etiology are crucial for early treatment of epileptic seizures. This article provides a comprehensive summary of the pathogenesis, clinical features, diagnosis and treatment of epilepsy associated with organic acid metabolism disorders. Furthermore, relevant literature has also been reviewed to assist clinicians in the diagnosis of cases characterized by the coexistence of multisystemic symptoms and epileptic manifestations.

## Background

Epilepsy is a chronic neurological disease caused by various factors that lead to excessive neuronal discharges in the brain, resulting in recurrent, episodic, and transient central nervous system malfunctions [1]. Organic acid metabolism disorders have been linked to the occurrence of epileptic seizures. These disorders comprise a series of conditions in which defects in enzymes in the biochemical pathways cause the toxic accumulation of several metabolites in the body. The disorders’ clinical manifestations of these disorders are typically nonspecific and may present with multisystemic symptoms [2], including psychomotor retardation, hypotonia, microcephaly, and metabolic acidosis. Patients with these disorders may experience various types of seizures, such as epileptic spasms [3], myoclonic seizure [4], generalized tonic-clonic seizure (GTCS) [5]. Clinical diagnosis of organic acid metabolism disorders can be assisted by gas-liquid chromatography examination of organic acids and their derivatives in urine and blood, and other auxiliary diagnostic methods include electroencephalogram, cranial nuclear magnetic resonance, enzyme analysis, and genetic testing, etc. Long-term treatment can be applied to limit the intake of precursor substances that lead to the accumulation of organic acids and promote the metabolism of the accumulated products. In addition, acute metabolic disorders often occur due to infections or stress, which can even be life-threatening in severe cases. Therefore, reversing the catabolic state and accelerating the excretion of metabolites should be an emergency necessity for clinical treatment [6].

This paper provides a comprehensive overview of the pathogenic mechanisms, clinical features, diagnosis and treatment of epilepsy associated with organic acid metabolism disorders, and also reviews the relevant literature to improve the diagnosis of cases with coexisting multisystemic symptoms and epileptic manifestations in clinical practice.

## Disorders of organic acid metabolism

### Methylmalonic acidemia

**Fig. 1:**
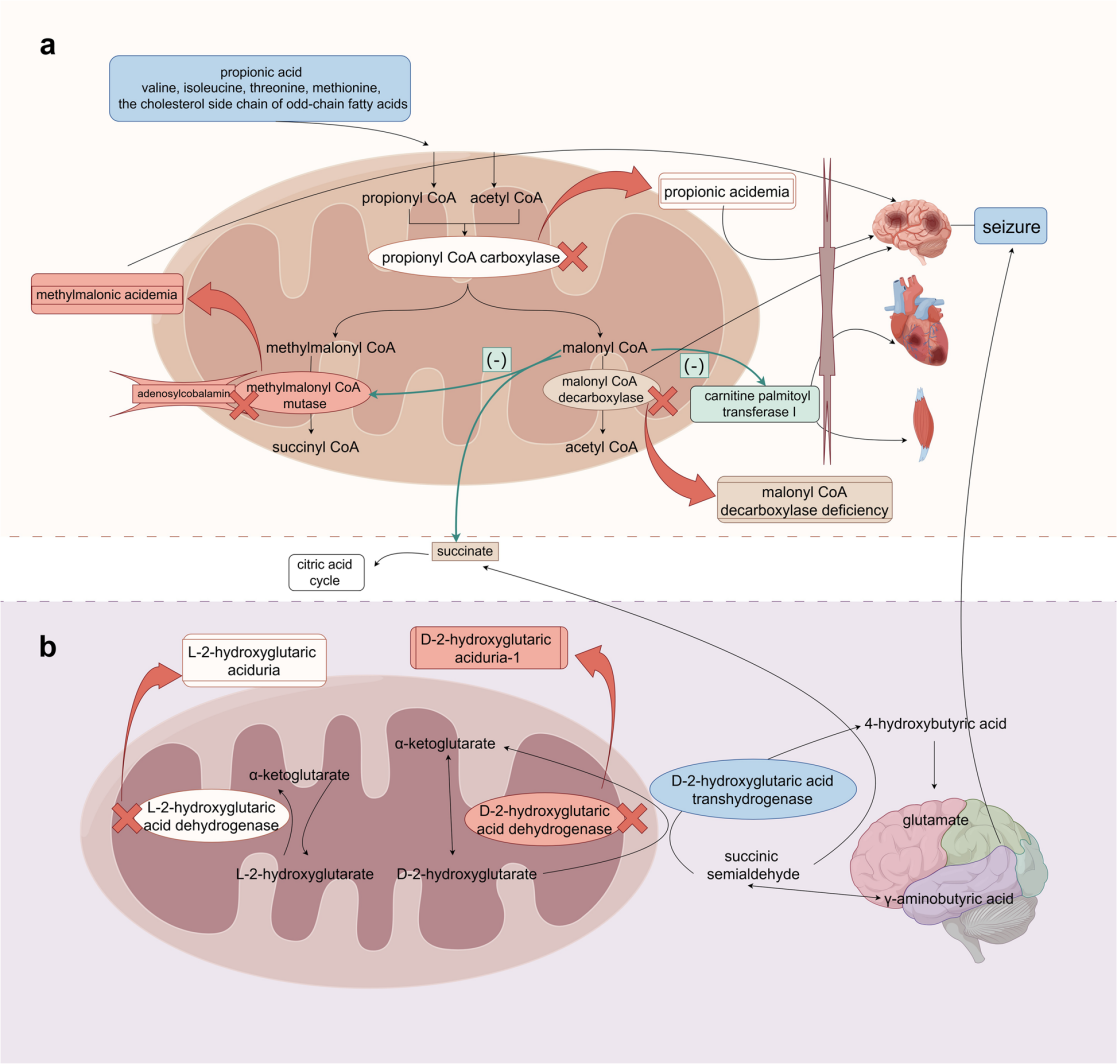
**a** Metabolic map of methylmalonic acidemia, propionic acidemia, and malonyl-CoA decarboxylase deficiency; **b** Metabolic map of L-2-hydroxyglutaric aciduria and D-2-hydroxyglutaric aciduria -1

Methylmalonic acidemia (MMA) is an autosomal recessive disorder caused by a deficiency in methylmalonyl coenzyme A (CoA) mutase (MUT), which is encoded by the *MUT* gene. MUT plays a vital role in converting methylmalonyl CoA to succinyl CoA in the catabolism of propionic acid, and which requires the participation of adenosylcobalamin (vitamin B12, Adocbl) as a cofactor in this process [7] (Fig. 1a). Genetic variations in the *MUT* gene lead to the absence of methylmalonyl CoA mutase, resulting in a blockage of the propionate metabolic pathway. As a consequence, methylmalonyl CoA is converted into toxic substances such as methylmalonic acid, resulting in a range of clinical manifestations associated with varying degrees of severity in MMA. The pathophysiological mechanism of epilepsy caused by methylmalonic acidemia is related to the activation of N-methyl-D-aspartate (NMDA) receptors. The accumulation of toxic substances leads to oxidative damage to cells and a decrease in Na^+^-K^+^-ATPase activity, which results in the activation of NMDA receptors, neuronal depolarization, and clinical symptoms such as seizures.

Epilepsy induced by MMA is related to reduce glutamic acid decarboxylase (GAD) activity [8]. The disease can present with symptoms affecting multiple systems, including lethargy, vomiting, shortness of breath, symptoms of metabolic acidosis, developmental delay, epilepsy, etc. Under the condition of stress reactions such as fever, infection, or increased metabolic stress, patients with methylmalonic acidemia may manifest symptoms of acute metabolic decompensation such as impaired level of consciousness, hypotonia complicated by hyperammonemic encephalopathy, respiratory distress, acidosis, etc. In 2011, Zhao et al. reported several cases of MMA patients with epilepsy as a manifestation [9]. These patients presented with various types of seizures, including generalized tonic-clonic seizures, focal seizures, tonic seizures, myoclonic seizures, epileptic spasms, and status epilepticus. Waking and sleeping electroencephalography (EEG) were performed on the reported patients, revealing different EEG manifestations of seizures complicated by methylmalonic acidemia. These EEG findings include background rhythm slowing, focal or multifocal epileptiform discharges, generalized epileptiform discharges, high-amplitude dysrhythmia, generalized voltage suppression, and atypical bursting-suppression [9, 10]. Biochemical tests for MMA may show elevated levels of blood propionylcarnitine and/or elevated propionylcarnitine to acetylcarnitine ratio, and markedly elevated urinary methylmalonic acid and methyl citrate [11]. Patients with this disease may exhibit various abnormalities on cranial magnetic resonance imaging (MRI), including cerebral atrophy, white matter abnormalities, hypoplasia of the corpus callosum, bilateral basal ganglia abnormalities, and symmetric necrosis of the bilateral pallidum.

Genetic variants in the *MUT* gene, causing MMA, can be divided into two types: (Mut-), a partial genetic variant in the *MUT* gene that results in low to moderate residual MUT activity in the presence of high concentrations of Adenosylcobalamin (AdoCbl), and (Mut0), a complete genetic variant in the *MUT* gene resulting in virtually undetectable MUT activity [12]. The latter type, Mut0, dose not respond to vitamin B12 treatment. MMA can be treated with cobalamin injections on an individual basis, and long-term treatment includes the use of special formulas limiting the precursor substances of propionic acid (methionine-, valine-, threonine-, and isoleucine-free): high-calorie diets are provided to support growth and development, carnitine supplementation is recommended to promote organic acid excretion, and application of antibiotics can be used to reduce the intestinal flora. In cases of acute metabolic failure in MMA patients, prompt emergency treatment should be carried out to minimize the damage to the central nervous system. This may involve interventions such as glucose and electrolytes supplementation, carnitine supplementation to promote the excretion of organic acids and correction of metabolic disorders. Chronic organ damage, particularly renal damage, may occur in patients with MMA. In severe cases, renal transplantation and liver transplantation may be necessary for the management of the condition [11].

### Propionic acidemia

Propionic acidemia (PA) is an autosomal recessive inherited metabolic disorder caused by genetic variants in the *PCCA* or *PCCB* genes, leading to propionyl CoA carboxylase (PCC) deficiency (OMIM #606054). PCC plays a crucial role in the propionic acid metabolic pathway by converting propionic acid and its precursors (valine, isoleucine, threonine, methionine, and the cholesterol side chain of odd-chain fatty acids) into propionyl CoA (Fig. 1a). When there are genetic variants in the *PCCA* or *PCCB* genes, propionyl CoA carboxylase is defective. As a result, propionic acid and its metabolic precursors accumulate, leading to the production of toxic metabolites such as 3-hydroxypropionic acid and methyl citrate, contributing to the nonspecific clinical manifestations and severity in PA.

PA is characterized by a variety of neurological symptoms, such as ataxia, epilepsy, and so on [13]. The pathophysiological mechanism of epilepsy caused by propionic acidemia is similar to that of methylmalonic acidemia. In typical neonatal cases of PA, symptoms usually appear within hours to days after birth and are often accompanied by acute metabolic disturbances, poor general condition, feeding difficulties, vomiting, dehydration and temperature instability. Neurologic involvement can present as hypotonia or hyperactivity, irritability, lethargy, or seizures. Delayed-onset propionic acidemia can occur at any age: infancy, childhood, or even later. Symptoms in these cases may include growth retardation, intellectual disability, or acute metabolic disorders caused by metabolic stress, mainly manifested as infections, but also with systemic complications, such as epilepsy, ketoacidosis, recurrent vomiting, leukopenia, or cardiac muscle disease [11].

The clinical diagnosis of PA involves several diagnostic methods. Urine gas chromatography-mass spectrometry analysis suggests increased 3-hydroxypropionic acid and methylcitric acid, and blood tandem mass spectrometry analysis of dried blood spots of acylcarnitine suggests significantly elevated propionylcarnitine [14]. As for imaging, cranial MRI may reveal cerebral atrophy [15–18], delayed myelin formation [18] and various changes in the basal ganglia; proton magnetic resonance spectroscopy (MRS) may show decreased peaks of N-acetylaspartate and inositol, as well as increased levels of glutamine, reflecting the neurologic effects of hyperammonemia [13]. Seizures and EEG abnormalities are commonly observed in patients with PA and may be related to the neurologic effects of hyperammonemia and the heightened sensitivity of the basal ganglia to metabolic disturbances [6, 19]; EEG changes can be widespread or focal.

Currently, there is no specific treatment for propionic acidemia. The management of the condition primarily focuses on symptomatic and supportive treatment, including dietary restriction (restricting amino acids and odd-chain unsaturated fats as precursor substances for propionic acid), carnitine supplementation, and the use of metronidazole and other antibiotics to inhibit intestinal bacteria [20]. Liver transplantation can be taken into consideration if necessary. In addition, when acute metabolic disorders occur, treatment should be based on the principles of reversing catabolism and eliminating toxic metabolites, with glucose and fat milk supplementation to reverse catabolism, carnitine supplementation, and hemodialysis or peritoneal dialysis for the removal of toxic compounds. If hyperammonemia occurs, sodium benzoate/phenylacetic acid, N-carbamoylglutamic acid, and arginine can be applied to promote the urea cycle to counteract the hyperammonemia [21].

### Malonyl CoA decarboxylase deficiency

Malonyl CoA decarboxylase deficiency is a rare autosomal recessive disorder that affects organic acid metabolism. It is caused by a pure or compound heterozygous genetic variant in the *MLYCD* gene located on chromosome 16q24 [22]. The *MLYCD* gene encodes malonyl CoA decarboxylase (MCD), whose carboxyl terminus functions as a peroxidase, and which is involved in the conversion of malonyl CoA to acetyl CoA and carbon dioxide in mitochondria and peroxisomes [23] (Fig. 1a). Malonyl CoA is an important substance required in fatty acid metabolism, and cytoplasmic malonyl CoA can regulate the balance of fatty acid synthesis and oxidation by inhibiting carnitine palmitoyltransferase I (CPT1). The CPT1 subunit in the heart, skeletal muscle, and liver is inhibited by malonyl CoA [23], with heart and skeletal muscle being more sensitive to the regulation of this enzyme, and the brain-specific CPT1 subunit having a high affinity for malonyl CoA [24]. When the MCD enzyme is defective, it can lead to abnormal levels of malonyl CoA, which in turn can impact brain CPT1 and contribute to abnormal brain development.

Common clinical manifestations of malonyl CoA deficiency include psychomotor retardation, seizures, hypotonia, hypertrophic cardiomyopathy, metabolic acidosis, diarrhea, vomiting, and hypoglycemia; cardiomyopathy and metabolic acidosis may be associated with increased oxidation of accumulated malonyl CoA [25]. It has been suggested that patients with this disease may also exhibit refusal to eat, potentially due to the accumulation of malonyl CoA in the hypothalamus, where high levels of malonyl CoA can suppress appetite and feeding [26, 27]. Abnormalities in lipid metabolism may also be observed in individuals with MCD enzyme deficiency, reported cases have shown increased plasma triglyceride levels, abnormal fasting plasma lipoprotein profiles, and increased risk of celiac disease [28].

Biochemical tests may indicate elevated levels of malonate and methylpropionic acid in the urine. Increased excretion of methylpropionic acid is associated with inhibition of methylmalonyl CoA by malonyl CoA [28]. Organic acid metabolism disorders such as increased malonyl carnitine and succinic acid, as well as metabolic acidosis and hypoglycemia may also occur. Enzymatic examination can be performed by measuring the decreased activity of mitochondrial MCD enzyme in fibroblasts. Genetic testing is also valuable in the diagnosis of this disease. Cranial MRI may reveal cerebral atrophy, loss of white matter around the ventricles, cortical thickening, nodular ectopic, thinning of the corpus callosum, and ventricular dilatation [29]. Cranial CT may show frontotemporal lobe atrophy, bilateral basal ganglia hypodensity changes, and other abnormalities [30].

When consuming a high-fat and low-carbohydrate diet, patients with MCD enzyme deficiency may experience worsened clinical symptoms, such as hypoglycemia, metabolic acidosis, and increased urinary excretion of organic acids, etc. Therefore, it is important to manage these patients by providing a low-fat, high-protein diet and carnitine supplements. This approach can help reduce the excretion of urinary organic acids to normalize, and reduce hypoglycemic episodes. Hospitalization is recommended for these patients who have increased metabolic levels due to infection or fever [31].

### Creatine deficiency syndrome

Creatine is a nitrogenous organic acid that serves as the main source of energy for the brain, heart and muscles [32]. It is obtained from the diet, while in vivo, it is primarily synthesized by the kidneys, pancreas, and liver. The endogenous synthesis pathway involves two enzymes: arginine glycine acyltransferase (AGAT) and guanidinoacetic acid methyltransferase (GAMT) [33]. Genetic variants in the genes encoding either of these enzymes can lead to creatine deficiency syndrome (CDS), which is neurologically manifested as cerebral creatine deficiency syndrome (CCDS), with genetic variants in the genes encoding the enzymes leading to the syndromes known as CCDS2 and CCDS3, respectively. In the supply of energy to the brain, creatine needs to pass through the sodium- and chlorine-dependent creatine transporter (CRTR) in the blood-brain barrier, which encodes the gene *SLC6A8*, and the brain produces less creatine. The mutation of the transporter gene can also lead to the occurrence of CCDS, called CCDS1. The main organ involved in primary creatine deficiency is the brain, and 1H-MRS may show a significant reduction or absence of the creatine peak. Common clinical manifestations present with neurological symptoms, such as developmental delay, intellectual disability, delayed speech, autistic behavior, and/or varying degrees of epilepsy, ranging from occasional to intractable seizures. Other manifestations include failure to thrive, low muscle mass, hypotonia, and movement disorders (primarily extrapyramidal) [34].

#### *SLC6A8* gene-related CDS and epilepsy

The *SLC6A8* gene is located on chromosome Xq28 and contains 13 exons spanning 8.5 kb of genomic DNA [35]. CCDS1, caused by genetic variants in the *SLC6A8* gene, is an X-linked inherited metabolic disorder with common clinical manifestations including mental retardation, severe speech disorders, epilepsy, movement disorders, and behavioral disorders. *SLC6A8* plays a crucial role in the utilization of creatine in the body.

To date, there have been more than 160 case reports of CCDS1 [36]. In 2020, Rostami et al. reported two cases of new variants of primary creatine deficiency syndrome [37].One of these cases involved CCDS with recurrent seizures and neurological deterioration caused by a genetic variant in the *SLC6A8* gene. The patient had seizures that began 2 months after routine vaccination, followed by recurrent seizures with different types (manifesting as convulsive seizures in the first month of life, followed by focal seizures), along with cognitive, verbal, and motor delays, as well as neurological deterioration. Notably, whole-head developmental delay/intellectual disability is a common feature of all cerebral creatine deficiencies (AGAT, GAMT, and CRTR) [34]. His brain MRI sequences (at ages 4, 5, and 6 years) showed significant brain atrophy, abnormal signaling changes in the deep white matter and dentate nucleus, as well as large subdural effusions in the frontoparietal and temporal lobe regions. After being diagnosed with CCDS1, the patient was initiated on creatine supplementation. However, the patient did not tolerate the drug dose well. After 6 months of adherence to creatine and arginine hydrochloride therapy, the patient’s Gross Motor Function Classification System score (GMFCS) improved. Despite this improvement, the most recent brain MRI still showed diffuse cerebral and cerebellar atrophy, and the patient’s overall prognosis remained poor.

In the case of CDS, X-linked chromium transporter protein deficiency is the most common, and there is currently no effective treatment. However, for some CCDS1 patients, high-dose supplementation therapy with L-arginine and L-glycine may reap the benefits of increased muscle mass, improved dyskinesia, decreased frequency of intractable seizures, and the ability to improve cognitive function to some extent [38].

#### *GAMT* gene-related CDS and epilepsy

The *GAMT* gene, located at 19p13.3, is 4.5 kb in length and encodes the aminotransferase that converts guanidinoacetic acid to creatine. Genetic variants in the *GAMT* gene cause CCDS2, an autosomal recessive inherited metabolic disorder. In addition to the common clinical manifestations shared with CCDS1, CCDS2 is characterized by specific behavioral disorders, including autistic behavior and self-mutilation [39].

To date, about 110 cases of CCDS2 have been reported [40]. In 2016, Pacheva et al. reported a case of GAMT deficiency (pure c.64dupG genetic variant) presenting with neurodevelopmental delay, rare epilepsy, behavioral disorders and mild hypotonia [41]. The patient’s first seizure occured at 10 months of age and presented as complex, febrile, generalized tonic-clonic spasms lasting for about 25 min. Subsequent seizures continued to be associated with complex febrile seizures occuring at the ages of 4 years and 10 months, 5 years and 9 months, and 5 years and 11 months. Myoclonic-like seizures occurred several times since the age of 2 and a half. Laboratory tests showed low creatinine and slightly elevated lactate in both plasma and urine. Imaging showed a normal MRI structure, but the magnetic resonance (MR) spectra indicated a lack of creatine spikes. The patient’s EEG performance deteriorated with age. At 1 year and 4 months of age, the EEG recordings during sleep stage I and II showed only a few instances of rhythmic dysrhythmic activity. At 2 years and 9 months of age, the awakening EEG showed significant slow rhythmic background activity. At 5 years and 9 months of age, the EEG during stage II of sleep showed 1–2 Hz high-amplitude dysrhythmic slow-wave activity with rare multifocal spikes or sharp waves in the δ and θ background activity, which is suggestive of modified hyporegulation. Neuromuscular examination revealed abnormal muscle tone, followed by awkward gaits and motor delays. Neuropsychological evaluation suggests severe mental retardation and mild to moderate autism. The above clinical manifestations and ancillary findings clarified the diagnosis of CCDS.

Supplemental therapy with creatine supplements, such as creatine monohydrate, can help replenish the brain’s creatine supply. While dietary therapy with ornithine supplementation and restriction of protein or arginine intake can help reduce the accumulation of neurotoxic guanidinoacetic acid and achieve a good improvement in the dyskinesia manifestations associated with CDDS2 [42].

#### *AGAT* gene-related CDS and epilepsy

The *AGAT* gene, also known as *GATM,* is located at 15q21.1. CCDS3, caused by genetic variants in the *AGAT* gene, is an autosomal recessive inherited metabolic disorder. It is important to note that AGAT deficiency is the only disorder that presents with additional myopathy, which may be related to guanidinoacetic acid (GAA) deficiency. As an alternative substrate for creatine kinase (CPK) [43], in GAMT and CRTR deficiency, GAA phosphate [44] provides an alternative high-energy reservoir to replace creatine phosphate, thus preventing myopathy. However, in AGAT deficiency, impaired GAA synthesis results in the absence of GAA phosphate formation, leading to complete depletion of muscle high-energy phosphate.

In laboratory tests, AGAT deficiency is characterized by low concentrations of GAA in body fluids, such as urine and plasma, whereas GAMT deficiency is characterized by high concentrations of GAA. CRTR deficiency in men is characterized by high urinary excretion, as evidenced by a high urinary creatine to creatinine ratio. Therapeutically, creatine supplementation is most effective in AGAT patients with myasthenia gravis and myopathy, and it can significantly improve or even normalize abnormal developmental scores [45].

### L-2 hydroxyglutaric aciduria

L-2hydroxyglutaric aciduria (L2HGA) is a rare, progressive, autosomal recessive disorder of organic acid metabolism. L-2-HGA-associated genetic variants inhibit the activity of L-2hydroxyglutaric acid dehydrogenase (L2HGDH), an important mitochondrial enzyme involved in the metabolic processes of glutamate and glutamine, which oxidizes the L-2-hydroxyglutarate into α-ketoglutarate [46] (Fig. 1b).

Common clinical manifestations of the disease include psychomotor retardation, macrocephaly, epilepsy, and signs of cerebellar abnormalities. Nearly 50% of patients with L2HGA exhibit macrocephaly, which may be the first symptom of L2HGA [47]. Macrocephaly occurs before the closure of the cranial sutures and is associated with brain edema [48]. Epilepsy is associated with the dysfunction of GABAergic system induced by L2HGA. About half of the patients have epilepsy and it usually occurs in the later stages of the disease [47, 49]. However, seizures or status epilepticus may also occur early in the course of L2HGA. These early seizures may be related to early subcortical white matter involvement, and similarly, severe subcortical involvement may be associated with recurrent generalized tonic-clonic seizures [50]. Regarding the types of seizures, some reports suggest that febrile convulsions, focal seizures and generalized tonic-clonic seizures can be challenging to control and require comprehensive treatment.

Işikay et al. reported a case of an 11-year-old female patient diagnosed with L2HGA in 2013, who presented primarily with generalized status epilepticus as the main clinical manifestation [50]. Upon admission, this patient exhibited abnormal deep tendon reflexes, indicating hyperreflexia. The neurological examination was repeated 2 days after the seizure, suggested mild cerebellar dysfunction, which was consistent with the clinical profile of L2HGA. Cranial MRI of the patient showed mild bilateral high signal lesions, mainly affecting the right frontal subcortical cerebral white matter, pallidum and dentate nucleus. Notably, the periventricular white matter, thalamus, and brainstem were unaffected, which is a typical MRI manifestation of L2HGA. The EEG recording of this patient within 12 h after cessation of status epilepticus was suggestive of a slowing of the left cephalic rhythm with frequent spike waves. MRS in patients with L2HGA may show a decrease in N-acetylaspartate and choline, along with an increase in inositol peaks [51].

Regarding the clinical diagnosis of L2HGA, it is necessary to test the levels of L-2 hydroxyglutaric acid in cerebrospinal fluid, blood and urine. Currently, the treatment options for L2HGA include a protein-restricted diet, application of antiepileptic therapeutic drugs for symptomatic treatment, and supplementation of riboflavin and carnitine (such as L-carnitine). Flavin adenine dinucleotide may activate the oxidation of L-2-HG to α-ketoglutaric acid, which further reduces the toxic effects of L-2-HG on the nervous system. L-carnitine, on the other hand, may combine with organic acids to form water-soluble metabolites and promote their excretion [52].

### D-2-hydroxyglutaric aciduria-1

D-2-hydroxyglutaric aciduria-1 (D-2-HGA1) is an extremely rare autosomal recessive inherited disorder of organic acid metabolism associated with genetic variants in the *D2HGDH* gene. This gene is located on chromosome 2q37.3 and is responsible for encoding D-2-hydroxyglutarate dehydrogenase, whose primary function is to convert D-2-hydroxyglutarate (D-2-HG) into α-ketoglutarate [53]. Another enzyme, known as D-2-hydroxyglutaric acid transhydrogenase, can also catalyze this conversion. It reduces succinic semialdehyde (SSA), a product of gamma-aminobutyric acid (GABA) catabolism, to 4-hydroxybutyric acid (Fig. 1b). Additionally, SSA can be converted to succinate for participation in the Krebs cycle. When genetic variations occur in the *D2HGDH* gene, D-2-hydroxyglutarate dehydrogenase is defective. As a result, there is an accumulation of D-2-hydroxyglutarate, which causes an increase in a variety of substances in the above reaction process. This accumulation may interfere with the metabolic pathway of the inhibitory neurotransmitter GABA. Furthermore, the accumulation of 4-hydroxybutyrate may have an effect on the excitatory neurotransmitter glutamate, which then have an influence on the nervous system. It has been suggested that the accumulation of D-2-hydroxyglutaric acid may contribute to impaired mitochondrial energy metabolism, increased oxidative stress, activation of N-methyl-D-aspartate (NMDA) receptors, and increased glutamate uptake, which may have toxic effects on cells and nerves [54], leading to the occurrence of epilepsy.

The clinical manifestations of D-2-HGA1 are highly variable. Common clinical manifestations include psychomotor retardation, seizures, hypotonia, cortical visual disturbances, cardiomyopathies and malformations, etc. Common types of epileptic seizures associated with D-2-HGA1 include GTCS, complex partial seizures, absence of concentration with myoclonic seizures, and late-onset epilepsy in adults and so on [55–57]. In the severe form, the clinical manifestations can present as infantile onset epileptic encephalopathy and cardiomyopathies, such as dilated cardiomyopathy. A reported case by Baker et al. [4] in 1997 described a patient with D-2-HGA1 who exhibited mild dysmorphic facial features, developmental delay, generalized hypotonia, epilepsy manifesting as myoclonic seizures, cortical blindness, and dilated cardiomyopathy. The child later developed congestive heart failure. EEG recordings conducted during wakeful and sleep periods at 6 months of age showed paroxysmal multispike and slow-wave activity in the right parietooccipital region, along with monorhythmic activity at a frequency of about 8 Hz in the left parietooccipital region. Normal visual evoked potentials and auditory brainstem evoked responses to flash stimulation could indicate the preservation of the visual pathway between the retina and the cortex. Muscle biopsy of this child showed a significant increase in glycogen in the muscle fibers and ultrastructural cylindrical helices in the submuscular region. EEG examination of patients with this kind of disease often shows multifocal paroxysmal spike activity and a high degree of dysrhythmia [58]. Biochemical tests demonstrated increased levels of D-2-hydroxyglutaric acid and citric acid cycle intermediates (such as succinic acid and lactic acid) in blood, urine, and cerebrospinal fluid, and increased protein concentration in cerebrospinal fluid [58] and γ-aminobutyric acid [59]. Neuroimaging examinations, such as cranial MRI, may show enlarged ventricles, subventricular cysts, thinning of the corpus callosum, frontal subarachnoid and subdural effusions, delayed maturation of the brain (such as delayed formation of myelin sheaths), and so on [4, 59].

Regarding the treatment of this disease, in 1993, Gibson et al. reported a patient with D-2-HGA1 who received high-dose phenobarbital, carnitine supplements, and a high-carbohydrate, low-fat diet, resulting in an effective reduction in the frequency of seizures. For the therapeutic aspect of stabilizing cardiac function in this disease, medications such as digoxin, furosemide and captopril can be applied. Several studies [4] have suggested that valproic acid may further exacerbate GABA pathway disorders and increase the concentration of 4-hydroxybutyric acid, which has a synergistic effect with the pathogenic mechanism of this disease. Therefore, valproic acid, gabapentin, and aminocaproic acid should be avoided in the treatment of D-2-HGA1, on the contrary, benzodiazepines such as chlordiazepoxide, which acts directly on GABAA receptors, can be considered for use (Table 1).

**Table 1:** The pathogenic genes, inheritance, seizure types, and treatment of disorders of organic acid metabolism associated with epilepsy

	Pathogenic gene	Inheritance	Types of seizures	Treatment
Methylmalonic acidemia	*MUT*	AR	GTCS, tonic seizure, clonic seizure, myoclonic seizure, focal seizures, epileptic spasms, etc.	ASM, Vit B12, special formula (free of methionine, valine, threonine, isoleucine)
Propionic acidemia	*PCCA /PCCB*	AR	GTCS, etc.	ASM, special formula (free of methionine, valine, threonine, isoleucine)
Malonyl coenzyme A decarboxylase deficiency	*MLYCD*	AR		ASM, a low-fat diet
Creatine deficiency syndrome				
CCDS1	*SLC6A8*	XLR	Focal seizures, epileptic spasms, etc.	ASM, supplemental therapy with large doses of L-arginine and L-glycine
CCDS2	*GAMT*	AR	GTCS, myoclonic seizure, etc.	ASM, creatine monohydrate and other creatine supplements, ornithine supplementation, restriction of protein or arginine intake
CCDS3	*AGAT*	AR		ASM, creatine monohydrate and other creatine supplements
L-2-hydroxyglutaric aciduria	*L2HGDH*	AR	GTCS, focal seizures, etc.	ASM, add riboflavin and carnitine (L-carnitine)
D-2-hydroxyglutaric aciduria-1	*D2HGDH*	AR	GTCS, myoclonic seizure, focal seizures, absence seizures, etc.	ASM (PB, benzodiazepines)
3-methylglutaconic aciduria	*HTRA2*	AR	Clonic seizure, etc.	ASM
Glutaric acidemia type 1	*GCDH*	AR	GTCS, atonic seizure, absence seizures, etc	ASM, special formula (lysine-free, arginine-rich), carnitine supplements
Fumaric aciduria	*FH*	AR	GTCS, epileptic spasms, focal seizures, clonic seizure, etc.	ASM (LCM)

*Abbreviations*: *AR* Autosomal recessive, *ASM* Anti-seizure medication, *GTCS* Generalized tonic-clonic seizure, *LCM* Lacosamide, *PB* Phenobarbital, *XLR* X-linked recessive

### 3-methylglutaric aciduria

3-methylglutaric aciduria is an autosomal recessive inherited metabolic disorder caused by defects in the *HTRA2* gene. The *HTRA2* gene is located at 2p13.1 and consists of eight exons spanning 3.8 kb. It encodes a serine protease named HtrA2 that is localized in the intermembrane space of mitochondria. When cells undergo apoptosis-stimulating stress, HtrA2 can either expose inhibitors of the apoptosis protein (IAP) binding motif to mediate cell death or mediate cell death through its serine protease activity [60]. Impaired function of HtrA2 leads to compromised mitochondrial function and increased lactate production [61].

Common clinical manifestations of 3-methylglutaric aciduria include seizures in the neonatal period or infancy, recurrent apnea leading to respiratory insufficiency, dystonia, and dysphagia [62, 63]. In 2018, Kovacs-Nagy et al. reported two cases of 3-methylglutaric aciduria caused by genetic variants in the *HTRA2* gene [61]. Patient 1 presented at birth with increased muscle tone in the peripheral muscles associated with pyramidal syndrome, and later experienced recurrent, intermittent clonic movements in the left lower extremity, which improved after intravenous phenobarbital administration. Additionally, respiratory insufficiency and severe dysphagia were observed. Patient 2 presented at birth with microcephaly, recurrent apnea, generalized hypotonia, dysphagia, and subsequent seizures. Laboratory investigations may reveal neutropenia, elevated 3-MGA in urine, and elevated lactate in blood and cerebrospinal fluid. In 2016, Mandel et al. reported one patient with 3-methylglutaric aciduria displayed thinning of the corpus callosum, enlarged lateral and third ventricles, and basal ganglia atrophy in cranial MRI. His MR spectra with shorter echo time (TE) (35 ms) revealed decreased N-acetylaspartate /creatine (NAA/Cr) ratios in white and gray matter, as well as increased choline and myo-inositol-to-creatine ratios in white matter, among others. Another patient had repeated abnormalities in the EEG, and at 4 months of age, the EEG presentation at the onset of seizure symptoms was consistent with myoclonic encephalopathy [63]. There is currently no specific regimen for 3-methylglutaric aciduria, and management primarily focuses on symptomatic supportive therapy, application of anti-seizure medications and assisted ventilation therapy.

### Glutaric aciduria I

Glutaric aciduria type 1 is an autosomal recessive disorder of organic acid metabolism caused by a double allele genetic variant in the *GCDH* gene located at 19p13.2 [64]. The *GCDH* gene is responsible for encoding the enzyme glutaryl CoA dehydrogenase (GCDH), which plays a crucial role in the degradation of lysine via the glutaryl CoA pathway. When lysine levels reach a desired concentration in the brain, the excess lysine is degraded by the GCDH. However, when a genetic variant occurs in the *GCDH* gene, resulting in the defective GCDH enzyme, glutaryl CoA is converted into glutaric acid and 3-hydroxyglutaric acid. Both of these byproducts are relatively impermeable as derivatives to cross the blood-brain barrier. Consequently, the accumulation of glutaric acid occurs, leading to the inhibition of GAD activity and a reduction in GABA synthesis. When the cumulative concentration of glutaric acid exceeds 2–3 times the plasma concentration, neurotoxicity and striatal neuron damage can occur, which may be related to excitotoxicity and oxidative stress [65, 66]. Glutaryl CoA also reacts with L-carnitine to synthesize glutaryl carnitine, increasing free CoA. When the accumulation of glutaryl CoA increases, excessive carnitine consumption occurs.

Common clinical manifestations of the disease include generalized dystonia, which can lead to complications such as speech disorders, mobility disorders and the occurrence of dysphagia, bradykinesia, macrocephaly, and seizures, etc. The seizures associated with this condition include tonic seizures followed by atonic and absence seizures [67] and GTCS [68] etc. Acute catabolic disorders can occur within the first year of life following upper respiratory tract infections or gastrointestinal tract infections. Some studies have shown that 80% of children under the age of 2 with this condition exhibit striatal degeneration [69]. Acute striatal necrosis can be divided into three stages [70].The first stage is cytotoxic edema in the basal ganglia, cerebral ischemia, and increased blood volume in the gray matter, which occurs within 24 h after motor deficits, and cranial MRI shows increased signal intensity on T2-weighted imaging of the shell nucleus and caudate nucleus [71], along with diffusion limitation [72]. The second stage is characterized by a decrease in striatal perfusion, glucose uptake, and development of secondary vasogenic edema, which typically occurs 4–5 days after the onset of symptoms. The third stage is the chronic phase of striatal atrophy, which can be characterized by persistent T2 hyperintensity in the nucleus accumbens and caudate nucleus on MRI [72]. Other neuroimaging tests in glutaric aciduria type 1, such as CT scans, may show “wing-like” and “bat-like” dilatation of the lateral fissure of the brain, early frontotemporal lobe atrophy, enlargement of the anterior temporal subarachnoid space, and hypodensity of the nucleus pulposus, etc. [73]. MRI may also show high density in bilateral basal ganglia, temporal lobe atrophy and extensive white matter hypodensity [74]. Biochemical tests for this disease may indicate elevated levels of 3-hydroxyglutaric acid and decreased excretion of C5-glutarylcarnitine in urine organic acid analysis [72, 75, 76]. Other biochemical tests may include hypoglycemia, metabolic acidosis, and decreased free carnitine levels. Enzymatic analysis can be performed to identify glutaryl CoA dehydrogenase defects in cultured fibroblasts, while genetic testing can aid in the diagnosis [72]. Prenatal diagnosis can be accomplished through amniocentesis and chorionic villus sampling to assess glutarate levels and GCDH enzyme activity [77, 78].

Long-term management of the disease involves dietary interventions aimed at restricting natural proteins and providing lysine-free, arginine-rich metabolic formulas. This approach helps minimize lysine metabolism across the blood-brain barrier. In addition, carnitine supplements, such as levocarnitine, can be used to increase carnitine levels in the body, which combines with glutaryl CoA to scavenge organic acids to control symptoms. Acute catabolic disorders may occur when infections are present. The key treatment is to reverse the catabolic state promptly, thus intravenous glucose, saline and L-carnitine can be injected to urgently stabilize the energy-fragile brain tissue, protect the nerves and prevent striatal necrosis [69, 79, 80].

### Fumarate hydratase deficiency

**Fig. 2:**
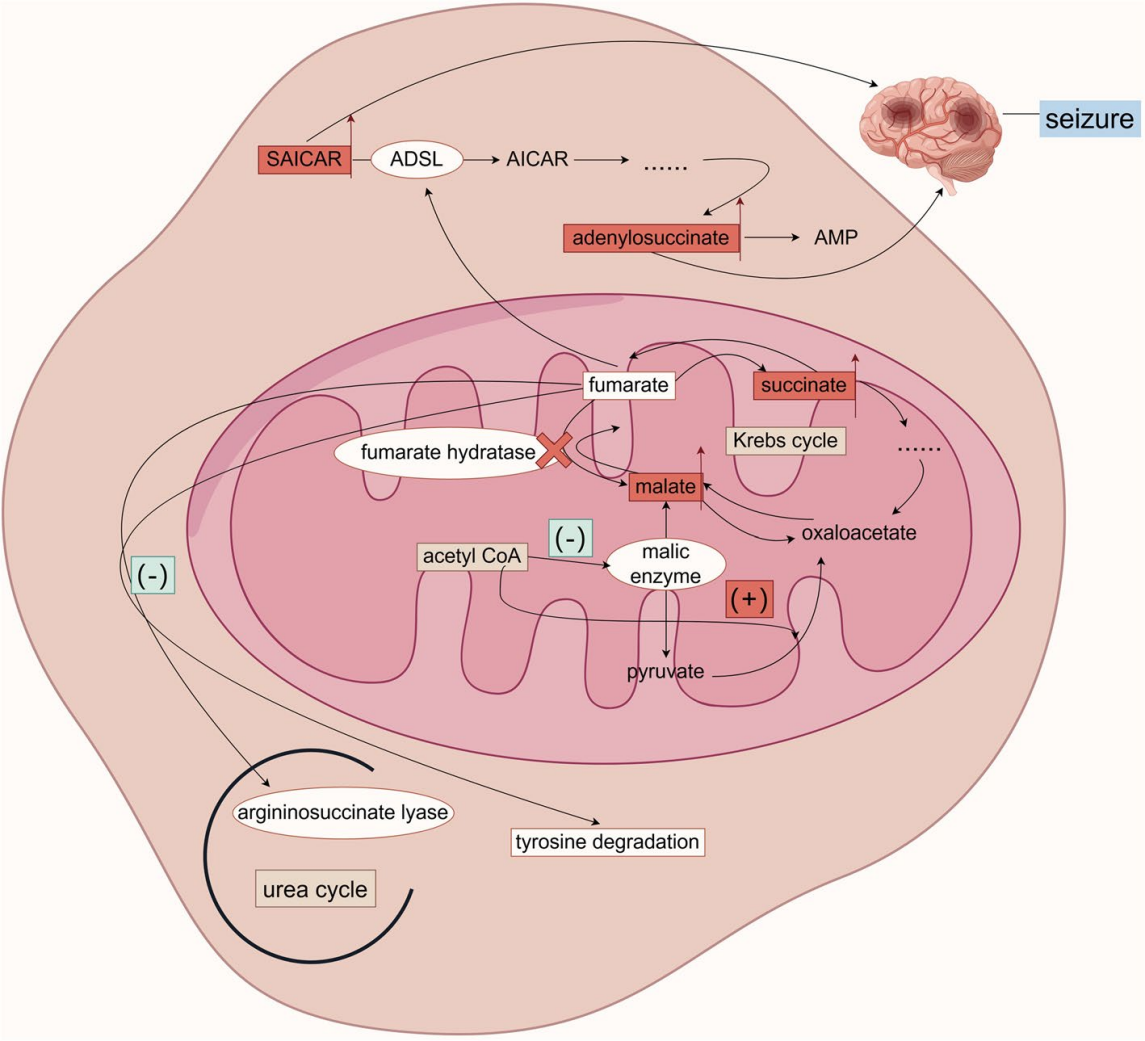
Metabolic map of fumarate hydratase deficiency Abbreviations: *ADSL* Adenylosuccinate lyase; *AICAR* Aminoimidazolecarboxamide ribose; *SAICAR* Succinylaminoimidazolecarboxamide ribose

Fumarate hydratase deficiency (FMRD) is a rare autosomal recessive disorder of organic acid metabolism caused by a genetic variant in the *FH* gene, locating on chromosome 1q42.1 [81]. This gene encodes fumarate hydratase (FH), which catalyzes the conversion of fumarate to malate. The mitochondrial FH enzyme is involved in the citric acid cycle, and the cytoplasmic FH enzyme is involved in the urea cycle, tyrosine catabolism, and purine de novo synthesis (Fig. 2). When FH is genetically variated, fumarate hydratase becomes defective, leading to fumarate accumulation increase, affecting a variety of metabolic pathways associated with it.

The common clinical manifestations of FMRD typically present as infantile encephalopathies in the neonatal period and early stages of life, such as poor feeding, growth retardation, hypotonia, lethargy, and epileptic seizures, etc. The common types of seizures may include epileptic spasms [3], status epilepticus, focal seizures secondary to generalized seizures, and in later stages of the disease, epileptic seizures may be manifested as myoclonic seizures and GTCS [5], etc. Facial abnormalities and microcephaly are also common features of the disease. In the citric acid cycle, increased fumarate and succinate levels enhance malic enzyme activity and increase the conversion of pyruvate to malate [82], which in turn inhibits the conversion of pyruvate to oxaloacetate when there is an accumulation of acetyl CoA, further increasing the accumulation of malic acid [83]. Fumaric acid accumulation also inhibits the activity of adenylosuccinate lyase (ADSL) in the purine de novo synthesis pathway, resulting in an increase in succinylaminoimidazolecarboxamide ribose (SAICAR) and adenosine succinate, which can cause damage to the central nervous system [84]. The accumulation of fumaric acid may also have an inhibitory effect on argininosuccinate lyase in the urea cycle, which affects the urea cycle and increases the amount of citrulline in biochemical tests. In addition, fumaric acid may affect the catabolism of tyrosine and inhibit the enzyme activity therein, which ultimately leads to an elevation of tyrosine and so on in biochemical tests [85]. Cranial MRI in patients with FMRD may show enlarged ventricles and subarachnoid spaces [86], abnormally high signal intensity in the periventricular white matter, cerebral atrophy, delayed formation of myelin sheaths, thinning of the corpus callosum, and an abnormally small brainstem [87]. The EEG of patients with this disease may show a high degree of dysrhythmia [88], 2–3 Hz generalized spike activity, with bifrontal lobe predominance [5], and multifocal spike-wave discharges followed by dysrhythmia [3]. Severe abnormalities in brainstem auditory evoked potentials [86] may also be present. Other diagnostic tests for FMRD include measuring FH enzyme activity in fibroblasts or leukocytes and genetic testing, etc.

Since there is no specific treatment for FMRD, most interventions focus on managing symptoms. Children with FMRD may experience drowsiness, aspiration and poor feeding, which can be addressed by using gastrostomy tubes. Physical therapy can be applied to alleviate the contractures and improve mobility. Anti-seizure medications (ASMs) are commonly used to manage seizures associated with FMRD. Grocott et al. [5] in 2019 reported a patient with FMRD developed seizures in the late course of the disease. After application of several first-line ASMs without efficacy, the frequency of seizures was significantly reduced by the addition of lacosamide. Infantile spasticity, which is often resistant to ASMs, may be treated with ACTH, and it should be noted that ketogenic diet therapy is contraindicated for epilepsy treatment in this disease.

## Conclusions

Organic acid metabolism disorders usually arise from enzyme deficiencies involved in amino acid degradation, resulting in the accumulation of organic acid in the brain and other tissues. This accumulation leads to the occurrence of a series of clinical symptoms, the symptoms can usually appear in infancy. However, the clinical heterogeneity of this type of disease can vary significantly, including different types of seizures. Clinically suspected seizures may present as abnormal movement, which can be related to extrapyramidal movement disorders related to basal ganglia injury. Therefore, the definite diagnosis of seizures needs to be differentiated from involuntary movement disorders related to basal ganglia injury. Avoiding misdiagnosis of patients with similar symptoms is essential for early identification and symptomatic treatment.

In clinical practice, it is important to pay close attention to patients presenting with seizures and multi-system symptoms suggestive of metabolic epilepsy. Some of the metabolic disorders can be diagnosed at the early stage of the disease based on relevant examinations. Early clinical intervention can be performed to delay the progression of the disease. Some disorders of organic acid metabolism can be treated by limiting the intake of precursors. However, it is important to note that the current treatment options for inborn errors of metabolism, especially organic acid metabolism disorders, are primarily symptomatic and lack of specific treatments targeting the underlying etiology and metabolic disorders. Future research holds the potential to bring about advancements in the field and provide more effective and targeted treatments for patients with metabolic epilepsy.

## Data Availability

Not applicable.
